# Pharmacokinetics and metabolomics investigation of an orally modified formula of standardized *Centella asiatica* extract in healthy volunteers

**DOI:** 10.1038/s41598-021-86267-2

**Published:** 2021-03-25

**Authors:** Phanit Songvut, Pajaree Chariyavilaskul, Phisit Khemawoot, Rossarin Tansawat

**Affiliations:** 1 Department of Pharmacology and Physiology, Faculty of Pharmaceutical Sciences, Chulalongkorn University, Bangkok, Thailand; 2 Translational Research Unit, Chulabhorn Research Institute, Bangkok, Thailand; 3 Clinical Pharmacokinetics and Pharmacogenomics Research Unit, Department of Pharmacology, Faculty of Medicine, Chulalongkorn University, Bangkok, Thailand; 4 Chakri Naruebodindra Medical Institute, Faculty of Medicine Ramathibodhi Hospital, Mahidol University, Samut Prakarn, Thailand; 5 Preclinical Pharmacokinetics and Interspecies Scaling for Drug Development Research Unit, Chulalongkorn University, Bangkok, Thailand; 6 Department of Food and Pharmaceutical Chemistry, Faculty of Pharmaceutical Sciences, Chulalongkorn University, Bangkok, Thailand

**Keywords:** Pharmacokinetics, Metabolomics

## Abstract

The formula of a standardized extract of *Centella asiatica* (ECa 233) was modified to improve its dissolution, with implications for pharmacokinetics and metabolomic profile. This study aimed to understand the resultant changes in disposition kinetics of ECa 233 and alterations to human metabolome after oral administration. This study was a two-sequence of dosages (250 and 500 mg), with an open-label phase I clinical trial. The modified formula was administered in single and multiple doses to twelve healthy Thai volunteers. The major parent compounds, madecassoside and asiaticoside, were rarely absorbed, instead undergoing biotransformation into active metabolites, madecassic acid and asiatic acid with possibility to be eliminated via fecal route. Increasing the dose of ECa 233 resulted in significantly greater plasma levels of those active metabolites, with accumulation of asiatic acid after multiple oral administration for seven days. Examining the impacts of accumulation behavior on metabolomics, the study traced changes in levels pre- and post-dose of five relevant human metabolites. Administration of ECa 233 was found to be significantly associated with an increase of choline, an endogenous metabolite with documented benefits for learning and memory. Therefore, ECa 233 may be useful in mitigating cognitive impairment, through its role in modulating human metabolites.

## Introduction

*Centella asiatica* (Linn.), commonly known as Asiatic pennywort or Gotu kola, belonging to the family Apiaceae*,* has been widely used in traditional medicine and possesses well-documented neuroprotective effects^1^. A standardized extract of *C. asiatica* was fully researched and developed as ECa 233 and was then characterized to contain more than 81% of triterpenoid glycosides^2^. Recent reports have shown the tolerability of ECa 233 in preclinical toxicology studies with NOAEL ≥ 1000 mg/kg/day in rats^3^. Pretreatment with 10 mg/kg of ECa 233 in mice was effective in mitigating the Alzheimer-like cognitive deficits induced by β-amyloid peptide^4,5^. Hence, ECa 233 has a potential to become a phytomedicine candidate for human use in terms of improving patients’ cognitive function.

Pharmacokinetics profiles of ECa 233 were first determined in a rat model^6^. Later, 250- and 500-mg capsules of ECa 233 were reported to be well tolerated in a phase 1 study in healthy subjects^7^. The parent compounds of ECa 233, which are MDS and ASS, were extensively biotransformed into active metabolites, MDA and ASA, respectively^7^. Most of the pharmacokinetic parameters of ECa 233’s parent compounds and active metabolites were illustrated; however, data about excretion and metabolomics are still limited^7^. To date, information in regard to the metabolome may directly reflect changes in the physiological state after oral administration of a specific drug and has been applied in the process of identifying biomarkers in metabolic disorder-involved diseases^8^. Understanding the associations between changes in the levels of candidate endogenous metabolites and the metabolite-related biomarkers of neurodegenerative disease^9^ is the way to support the next step in the use of ECa 233 in clinical practice.

A previous study found that ECa 233 had limited dissolution of formulation, leading to a restriction of its oral bioavailability^7^. Hence, the modified formula to improve oral bioavailability of ECa 233 was developed and was used in this clinical study. This research aimed to investigate the clinical pharmacokinetics profile and excretion pathway including metabolomic profiles of the modified-formula ECa 233 capsules in healthy Thai volunteers.

## Materials and methods

### Chemicals

The standardized *C. asiatica* extract is a white to off-white powder that contains the triterpenoid glycosides MDS (53.1%) and ASS (32.3%) as determined by the LC–MS/MS method. For this clinical study the extract was provided by Siam Herbal Innovation Co., Ltd (Thailand) (Lot number MRA 0816001). The modified formula of ECa 233 capsules (Lot number 18EC003005) was manufactured by Pharma Nueva Co., Ltd. (Thailand) under Good Manufacturing Practice quality standards. The appropriate dosage of *C. asiatica* extract used in this clinical study was determined by calculating the first dose in humans (FIH dose)^10^. The estimated maximum recommended starting dose (MRSD) was converted from the human equivalent dose (HED)^7^ based on the results of safety and pharmacological studies of ECa 233.

For LC–MS/MS analysis, methanol (HPLC grade) was obtained from Merck (Germany). The internal standards of glycyrrhizin (purity > 90%) and glycyrrhetinic acid (purity > 98%), and the reference standard of ASS (purity > 98.5%) were purchased from Sigma-Aldrich (Corp. USA). MDS (purity > 96.7%) was purchased from Chromadex, (Inc. USA). MDA and ASA, determined to be > 97.5% and 97.0% purity, respectively, were purchased from Wako Pure Chemical Industries, (Ltd. Japan).

For NMR analysis, deuterium oxide (D_2_O) was purchased from Cambridge Isotope Laboratories, (Inc. USA). Sodium hydrogen phosphate (Na_2_HPO_4_) for phosphate buffer preparation, sodium azide (NaN_3_) and trimethylsilyl propionic acid sodium salt (TSP) were purchased from Sigma-Aldrich, (Corp. USA).

### Ethical statement and informed consent

This study protocol was approved by the Institutional Review Board of Chula Clinical Research Center, Chulalongkorn University (IRB number: 479/61, COA number 850/2018, date of approval: 06/09/2018) and was registered in the Thai Clinical Trials Registry, a trial registration data set required by World Health Organization, (TCTR identification number: TCTR20180922001, date of first registration: 20/09/2018). The clinical study was conducted at Chula Pharmacokinetic Center, Maha Chakri Sirindhorn, Clinical Research Center, Faculty of Medicine, Chulalongkorn University, under the International Conference on Harmonization—Good Clinical Practice, and in accordance with the Declaration of Helsinki. All participants provided written informed consent for their participation prior to the start of the study.

### Eligibility criteria for participants

Twelve Thai healthy volunteers aged between 18 and 50 years were enrolled and recruited from March to April 2018. Inclusion criteria were BMI between 18.0 and 25.0 kg/m^2^, with normal medical histories, physical examinations, vital signs, and clinical laboratory tests. Participants were excluded if they smoked more than ten cigarettes/day, had a history of alcoholism or of moderate drinking (more than three drinks/day), were pregnant or breastfeeding, or planned to become pregnant during the study period. The number of subjects was defined according to the general guidelines for clinical pharmacokinetic studies, a concise guide used in clinical research^11^. Sample size determination was calculated for a 95% level of confidence according to the standard normal distribution *(Z*_*1-α/2*_ = *1.96)*, and a tolerated margin of error at 15% of the mean *(γ* = *0.15)*^12^.

### Study design and sample collection

**Figure 1:**
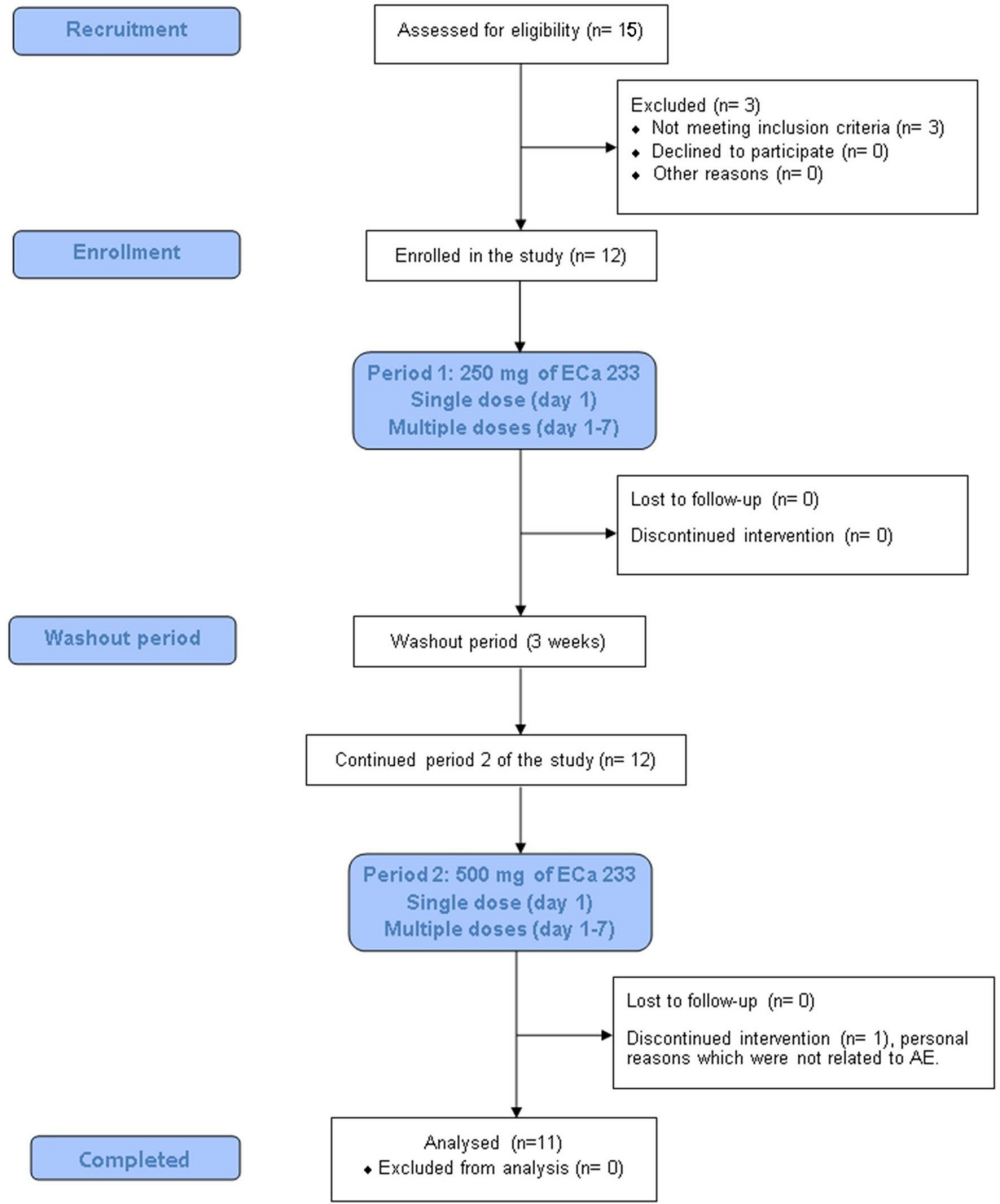
Flow diagram illustrates study design of the clinical trial.

**Figure 2:**
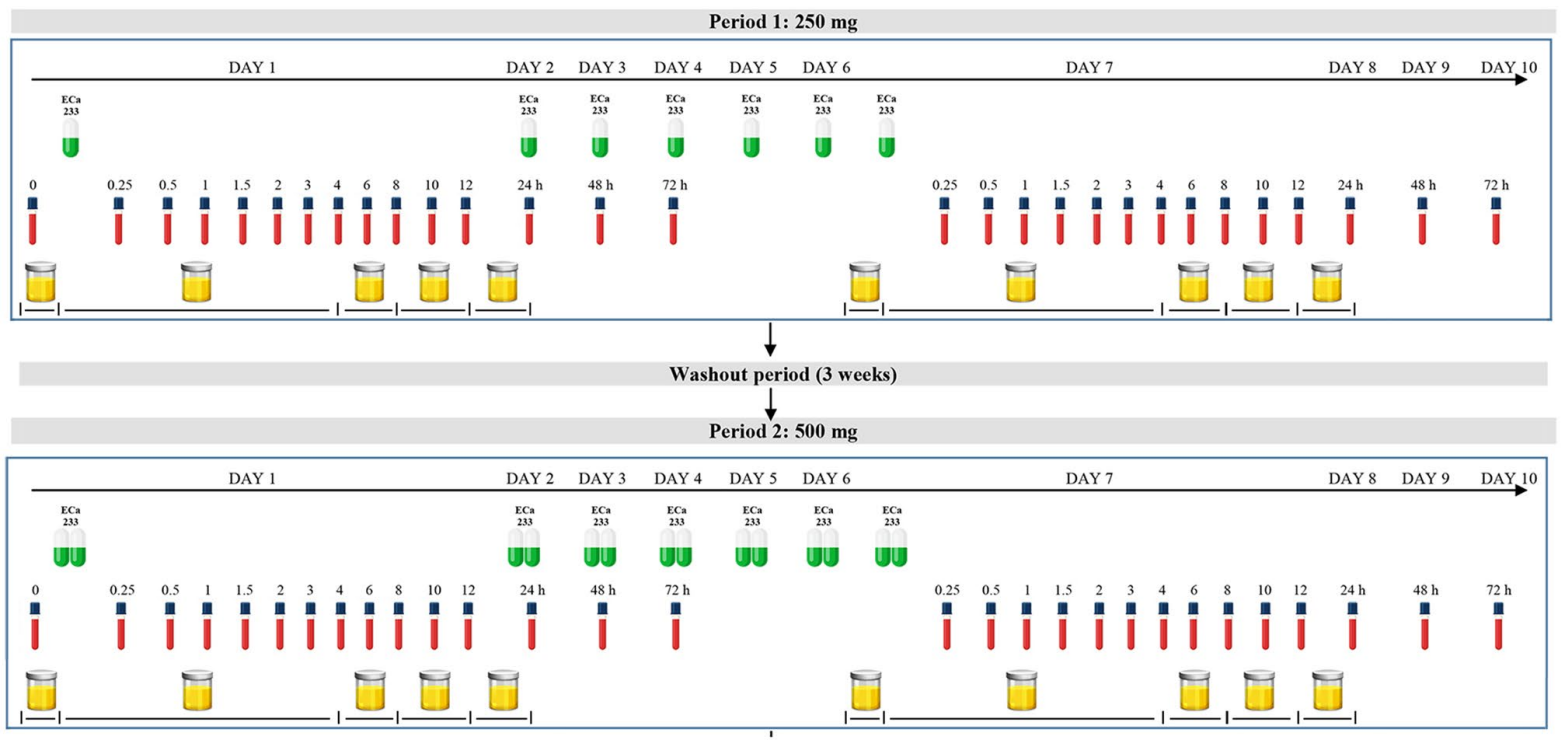
Timeline for oral administration, blood and urine collection.

This was a single-center, with an open-label, two-sequence of dosages (250 and 500 mg), two-periods of study (single and multiple dose) conducted in fasting condition (Fig. 1). In study period 1, all participants fasted overnight for at least 10 h before drug administration. The 250 mg of ECa 233 with 240 mL of drinking water were administered orally. Blood samples (3 mL) were collected from each individual at pre-dose (0 h) and 0.25, 0.5, 1, 1.5, 2, 3, 4, 6, 8, 10, 12, 24, 48, and 72 h post dose. All blood samples were centrifuged at 4 °C, 3200 × *g* for 10 min. Urine samples were collected at 0 h (the day prior to dosing) and 0–4, 4–8, 8–12, and 12–24 h after dosing. Feces were also collected from one of the volunteers for detecting the possible presence of any eliminated compounds. Feces samples were collected each time that a volunteer defecated on day 1–3, 4–6 and 7–9 during the study period. These samples were immediately fixed with methanol and prepared by our previously described method of protein precipitation. After a 3-week washout period, all participants continued period 2 of the study, where they received a 500-mg dose of ECa 233, and sample collection procedures remained consistent with period 1 (Fig. 2). Plasma, urine and fecal samples were then stored at − 80 °C until analysis.

For multiple oral dosing, participants continued to take 250 mg or 500 mg of ECa 233 every morning for 7 consecutive days. Then they had another set of blood and urine samples collected similar to the single dose study. The primary outcome was determination of pharmacokinetic parameters, including the profiles of the metabolites, and the secondary outcome was consideration of the safety profiles of ECa 233 after period 2 (end of the study).

### Sample preparation and pharmacokinetic analysis by LC–MS/MS

Sample preparation was performed by protein precipitation with two internal standards (glycerrhizin and glycerrhetinic acid) to correct the quantitative analysis of parent compounds and of their metabolites. Briefly, 200 µL of methanol was added followed by vortex mixing with 50 µL of each sample for 10 min and then centrifuged at 12,000 × *g*, 4 °C, 10 min. Quantitative determination of the parent compounds and their active metabolites in plasma and urine were carried out by using liquid chromatography tandem mass spectrometry (LC–MS/MS): LCMS-8060 (Shimadzu Corp. Japan) according to a previously validated and published bioanalytical method^7^. Chromatographic separation of the extracted sample was then performed on a Phenomenex Synergi 4u Fusion-RP (80A, 4 microns, size 50 × 2.0 mm) C18 column (column temperature = 40 °C) and eluted by two mobile phases: 0.2% formic acid in water (solvent A) and 100% methanol (solvent B), following gradients 0–0.5 min 10% B, 0.5–3 min 90% B, and 3–5 min 10% B, at a constant flow rate of 0.5 mL/min with 10 µL of injection volume. The MS/MS system was operated using an ESI source in negative mode with MRM. For *m/z* transition; MDS, ASS, MDA, ASA, glycyrrhizin, and glycyrrhetinic acid showed a precursor ion and product ion at 973.50/503.30, 957.55/469.00, 503.20/437.20, 487.60/409.25, 821.25/350.95, and 469.45/409.40, respectively (Supplementary Figs. 1S, 2S and 3S).

### Sample preparation and metabolomics analysis by ^***1***^H-NMR spectroscopy

Plasma samples at predose, 0.5, 1, and 2 h were used in the metabolomics part of the study. In brief, 400 µL of plasma were mixed with 400 µL of 0.142 M phosphate buffer in D_2_O solution (pH 7.4) containing 3 mM sodium azide (NaN_3_) used as a bacteriostatic reagent, and 0.08% trimethylsilylpropanoic acid (TSP) used as a chemical shift reference. The mixer was vortexed, sonicated at 25 °C for 15 min and then centrifuged at 13,000 × *g*, 4 °C for 10 min. Lastly, 600 µL of supernatant was transferred into a 5-mm diameter NMR tube for analysis.

All ^1^H NMR spectra of plasma samples were acquired at 600.13 MHz on a 600 MHz NMR spectroscopy (Bruker Avance III, Bruker Biospin, Germany) and were determined using a modified version of a previously published method^13^. The samples were measured at a temperature of 300 K using the presaturation sequence (64 scans). ^1^H NMR spectra were recorded by a standard one-dimensional (1-D) with the suppression of water signal, CPMG pulse. The spectra region was set to a range from − 1 to 10 ppm and the parameters were as follows: 64 K data points; relaxation delay of 2 s and mixing time (t_m_) of 100 ms.

### Data processing and statistical analysis of pharmacokinetics

Non-compartmental analysis was applied to determine pharmacokinetic parameters and performed by PK solutions software (version 2.0). AUC_(0-t)_ was calculated using the linear trapezoidal rule. t_½_ was calculated by *t*_*(1/2)*_ = *ιn*_*2*_*/k*_*el* ._ The apparent clearance (Cl/F) after oral administration was calculated as *Cl/F* = *Dose/AUC*, where F is the absolute oral bioavailability. The apparent volume of distribution (V_d_/F) was estimated using *V*_*d*_*/F* = *Dose/(AUC* × *k*_*el*_*)*. The C_max_ and T_max_ were determined directly from the observed concentration–time curve.

For statistical analysis in the pharmacokinetics study, all data were expressed as mean ± SD except for T_max_, which was expressed as median (IQR). The descriptive data were used to describe demographic characteristics and to summarize the continuous variables. Data processing was performed using STATA software (version 10.0; StataCorp, USA) and displayed in the graphical charts by using GraphPad Prism 8. A paired t-test or Wilcoxson Signed Rank Test was used to compare pharmacokinetics parameters between single and multiple doses. To determine statistically significant differences of the pharmacokinetics parameters of two dosages between 250- and 500-mg, Student’s t-test or Mann–Whitney U test was used where appropriate. A p-value of less than 0.05 was considered statistically significant.

### Data pre-processing, multivariate statistical analysis, and metabolite identification for metabolomics

All plasma spectra were pre-processed using TopSpin 3.1 software (Bruker, Germany) including baseline correction and TSP calibration at 0.00 ppm. The NMR spectra were subsequently transferred into MATLAB software (MathWorks, R2014a, USA). The spectral regions containing TSP peak (δ^1^H − 1 to 0.66 ppm) and water peak (δ^1^H 4.599 to 5.053 ppm) were omitted. The spectral alignment was then applied to adjust the position for all remaining peaks, and those aligned NMR spectra were imported into SIMCA 14.0 (Umetrics Umea, Sweden). In order to provide a general overview on clustering, data were initially analyzed by an unsupervised principal component analysis (PCA) with unit variance (UV) scaling. In considering the possible influences of ECa 233, a clear separation of suspicious metabolome changes between pre- (T_0_) and post-dose (T_0.5, 1, 2 h,_ on day 7) was further analyzed by OPLS-DA in MATLAB (R2014a) using the in-house developed scripts at Imperial College London. The OPLS-DA model was evaluated according to two parameters: R^2^X explained the fitness and Q^2^Y represented the predictive capability of the model. The permutation test was assessed by using 1000 times for the validation of all models involved in this study. The coefficient loadings plot of OPLS-DA models was used to indicate the significantly different metabolites as compared between pre-dose and post-dose. The permutation p-values were used to illustrate the level of differences that were considered as statistically significant at p-values less than 0.05.

For metabolite identification, plasma endogenous metabolites were determined using Chenomx NMR Suite 8.2 (Chenomx Inc., Canada), and were further justified in statistical total correction spectroscopy (STOCSY) using MATLAB software. In addition, the human metabolome database (HMDB) as well as the previously published assignments and in-house chemical shift databases were also used to support the metabolite identification in this study.

## Results

### Participants baseline clinical characteristics

All subject demographics and characteristics are summarized in Table 1. The age limit for study volunteers was 18–50 years; people in this age range were eligible to participate. The age of enrolled participants, however, was within a smaller range, from 21 to 38 years. One subject withdrew consent during the second period of the study due to personal reasons, which were neither ECa 233-related problems nor intolerable side effects. Therefore, there were eleven subjects that completed the study and remained for the per-protocol analysis.

**Table 1:** Subject Demographic and baseline characteristics of the study volunteers.

Demographic data	Baseline	ECa 233, 250 mg	ECa 233, 500 mg
**Gender, % (n)**			
Female		50% (n = 6)	45.5% (n = 5)
Male		50% (n = 6)	54.5% (n = 6)
Age^a^ (year)		26 [9.5]	26.5 [9.25]
Body mass index^b^ (kg/m^2^)	21.49 ± 1.66	21.37 ± 1.70	20.95 ± 1.58
Systolic blood pressure^b^ (mmHg)	110 ± 10	117 ± 7	115 ± 11
Diastolic blood pressure^b^ (mmHg)	65 ± 11	64 ± 6	59 ± 5
Body temperature (°C)	36.6 ± 0.4	36.3 ± 0.6	37.0 ± 0.1
**Clinical laboratory screening**^**b**^			
White blood cell (× 10^3^/µL)	6.58 ± 0.89	6.11 ± 1.29	6.74 ± 1.91
Red blood cell (× 10^6^/µL)	5.05 ± 0.31	4.84 ± 0.29	4.83 ± 0.42
Hemoglobin (g/dL)	13.6 ± 1.9	13.0 ± 1.7	13.0 ± 2.1
Platelet (× 10^3^/µL)	307 ± 69	299 ± 77	317 ± 85
Fasting blood glucose (mg/dL)	84.58 ± 3.99	87.25 ± 6.28	89.55 ± 6.42
Blood urea nitrogen (mg/dL)	13 ± 3	12 ± 4	11 ± 4
Creatinine (mg/dL)	0.72 ± 0.19	0.73 ± 0.20	0.79 ± 0.21
Albumin (g/dL)	4.48 ± 0.26	4.58 ± 0.23	4.45 ± 0.27
Total bilirubin (mg/dL)	0.70 ± 0.42	0.68 ± 0.37	0.60 ± 0.30
AST (U/L)	18 ± 5	23 ± 15	19 ± 5
ALT (U/L)	18 ± 9	19 ± 9	20 ± 14
Alkaline phosphatase (U/L)	52 ± 9	52 ± 9	52 ± 9
Total cholesterol (mg/dL)	173 ± 25	174 ± 21	172 ± 25
Triglycerides (mg/dL)	76 ± 46	77 ± 34	77 ± 48
HDL cholesterol (mg/dL)	55 ± 8	57 ± 8	56 ± 8
LDL cholesterol (mg/dL, calculated)	104 ± 22	101 ± 20	100 ± 24
Sodium (mmol/L)	137 ± 1	137 ± 1	138 ± 1
Chloride (mmol/L)	105 ± 2	107 ± 2	105 ± 2
Potassium (mmol/L)	3.9 ± 0.3	3.9 ± 0.3	4.2 ± 0.3
Calcium (mg/dL)	9.5 ± 0.4	9.7 ± 0.3	9.5 ± 0.4
Magnesium (mg/dL)	2.5 ± 0.1	2.4 ± 0.1	2.4 ± 0.2
Phosphorus (mg/dL)	3.5 ± 0.4	3.9 ± 0.5	3.9 ± 0.4

Decimal numbers were reported according to laboratory standard of Maha Chakri Sirindhorn Clinical Research Center, Faculty of Medicine, Chulalongkorn University.

*AST* aspartate aminotransferase, *ALT* alanine aminotransferase, *HDL* high-density lipoprotein, *LDL* low-density lipoprotein.

^a^Data is expressed as median [IQR].

^b^Data are expressed as mean ± SD, (n = 11–12).

### Pharmacokinetics

#### Pharmacokinetic profiles of the modified formula ECa 233

**Figure 3:**
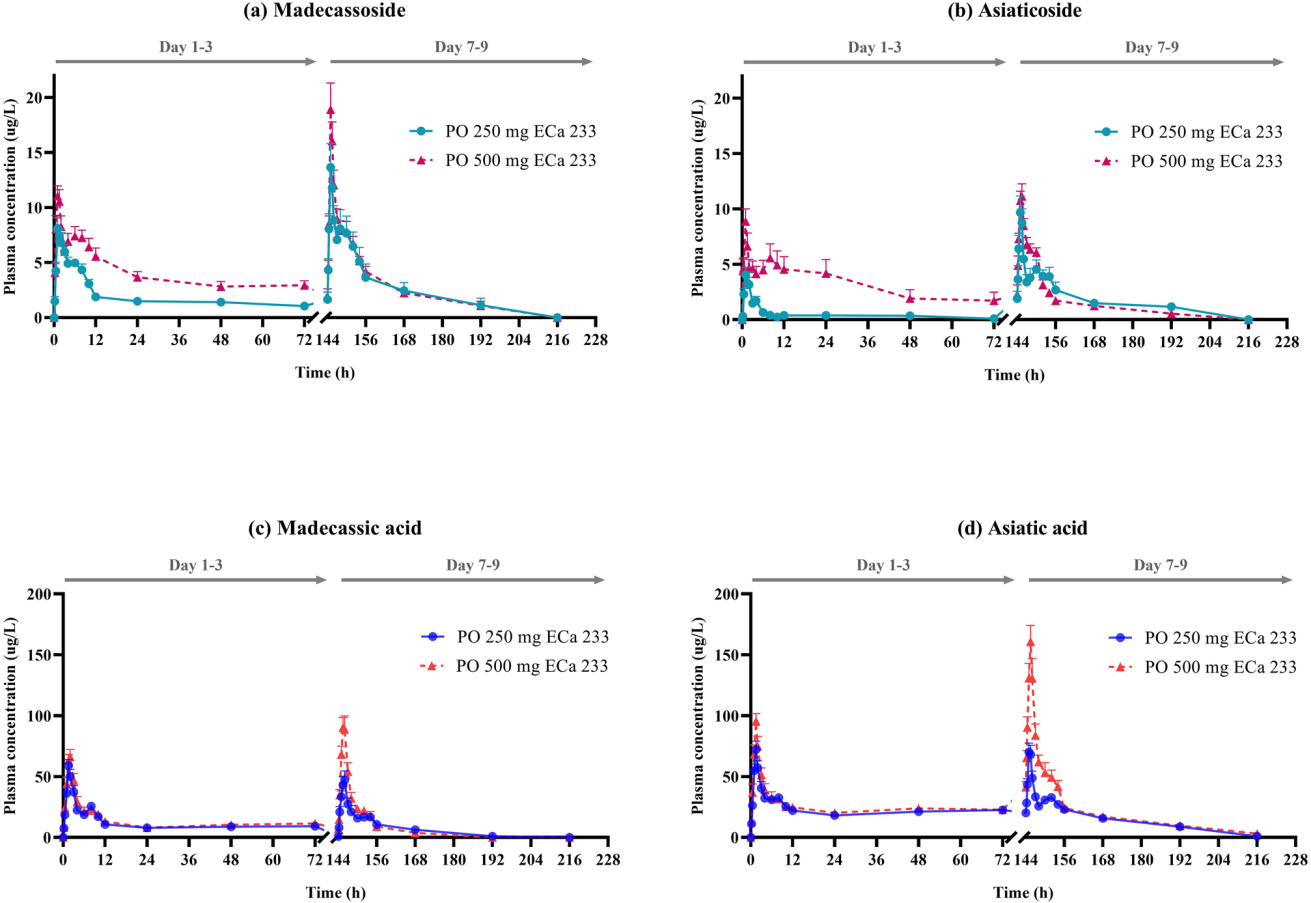
Mean plasma concentration–time profiles of the modified dissolution ECa 233; (**a**) madecassoside, (**b**) asiaticoside, (**c**) madecassic acid, and (**d**) asiatic acid, after single or multiple oral administration of 250 or 500 mg doses in healthy Thai volunteers. Data are presented as means ± SD (n = 11–12).

Pharmacokinetic profiles after oral administration of modified formula ECa 233 capsules are represented in plasma concentration–time curves of the parent compounds (MDS or ASS) and metabolites (MDA or ASA) (Fig. 3a–d). C_max_ of MDS or ASS was observed within 1 h after dosing, followed by the appearance of MDA or ASA that reached their C_max_ within 1–2 h. A notably higher C_max_ with a greater AUC was observed in both parent compounds and metabolite profiles of the modified formula of ECa 233 when compared to its previous formulation.

In considering a dose comparison between the 250 mg and 500 mg, the C_max_ of MDA and ASA was found to increase significantly; however, no significant changes were observed in T_max_ and t_1/2_ (Table 2). Likewise, the apparent volume of distribution (V_d_/F) and the apparent clearance (Cl/F) for MDA and ASA did not show significant differences when compared between the two dosage regimens.

**Table 2:** Clinical pharmacokinetic parameters of parent compounds (MDS and ASS) and their metabolites (MDA and ASA) following oral administration of the modified ECa 233 capsules in healthy Thai volunteers.

Parameters^#^	250 mg (n=12)				500 mg (n=11)			
	Parent compounds		Metabolites		Parent compounds		Metabolites	
	MDS	ASS	MDA	ASA	MDS	ASS	MDA	ASA
**Single dose oral administration, Day 1 (T**_**0-24 h**_**)**								
C_max_^a^ (µg/L)	9.57 ± 3.34	5.18 ± 3.43	66.09 ± 17.09	79.76 ± 19.28	12.73 ± 2.73*	9.81 ± 3.53*	72.26 ± 16.33	102.32 ± 20.76
T_max_^b^ (h)	1 [0.5]	1.25 [0.625]	1.5 [0.5]	1.5 [0]	1 [0.5]	1 [3.5]	1.5 [0.25]	1.5 [0.5]
AUC^a^ (µg^.^h/L)	75.38 ± 26.60	25.20 ± 29.15	410.35 ± 100.24	647.73 ± 117.41	141.16 ± 41.15*	176.59 ± 177.06*	476.02 ± 116.59	733.09 ± 149.90
**Multiple dose oral administration, Day 7 (T**_**0-72 h**_**)**								
Parameters	MDS	ASS	MDA	ASA	MDS	ASS	MDA	ASA
C_max_^a^ (µg/L)	15.34 ± 6.96	10.93 ± 4.60	61.38 ± 20.47	78.23 ± 26.42	23.00 ± 4.99	12.77 ± 2.87	99.78 ± 27.57*	170.35 ± 53.79*
T_max_^b^ (h)	1 [0.125]	1 [0.125]	2 [0.625]	1.5 [0.5]	1 [0.5]	1 [0.5]	1.5 [0.75]	1.5 [0]
AUC^a^ (µg^.^h/L)	162.27 ± 140.81	114.98 ± 54.12	437.49 ± 174.96	1013.55 ± 221.14	176.24 ± 72.68	106.82 ± 34.59	516.43 ± 156.64	1386.50 ± 332.86*
Vd/F^a^ (L/kg)	371.84 ± 386.32	155.55 ± 489.17	37.12 ± 28.53	10.53 ± 12.55	453.64 ± 376.55	458.14 ± 462.33	57.89 ± 24.35	23.39 ± 23.03
Cl/F^a^ (L/h/kg)	27.99 ± 23.88	17.09 ± 13.52	3.29 ± 1.70	0.74 ± 0.25	31.33 ± 16.15	30.48 ± 15.50	5.09 ± 1.64	1.11 ± 0.49
t_1/2_^a^ (h)	8.06 ± 5.62	10.37 ± 8.84	8.25 ± 5.85	9.86 ± 9.77	9.97 ± 9.39	9.35 ± 8.18	8.71 ± 5.46	14.13 ± 12.79

*C*_*max*_ maximum plasma concentration, *T*_*max*_ time to reach maximum concentration, *AUC* area under the plasma concentration–time curve from time zero to the last measurable concentration, *Vd/F* the apparent volume of distribution, *Cl/F* the apparent clearance, *t*_*1/2*_ elimination half-life.

^a^Data are expressed as mean ± SD; ^b^data is expressed as median [IQR]; * *p < 0.05* for significant differences.

^#^The desired pharmacokinetic parameters of Vd, Cl and t_1/2_ of both the parent compounds during single oral administration (day 1, 24 h) were not included in this table. These parameters are omitted for the parent compounds because the slope in their elimination phase did not end at 24 h post-dosing. Therefore, these parameters were calculated only for the metabolites.

Multiple oral dosing of the modified formula ECa 233 capsule resulted in greater plasma concentrations of ASA as compared to the single oral dosing. The AUC and C_max_ of ASA on day 7 were significantly higher than those on day 1 (*p* = 0.001 and 0.007, respectively), indicating that there was an accumulation of active metabolites after the repeated doses. The accumulation ratio of ASA was approximately 1.891.

**Figure 4:**
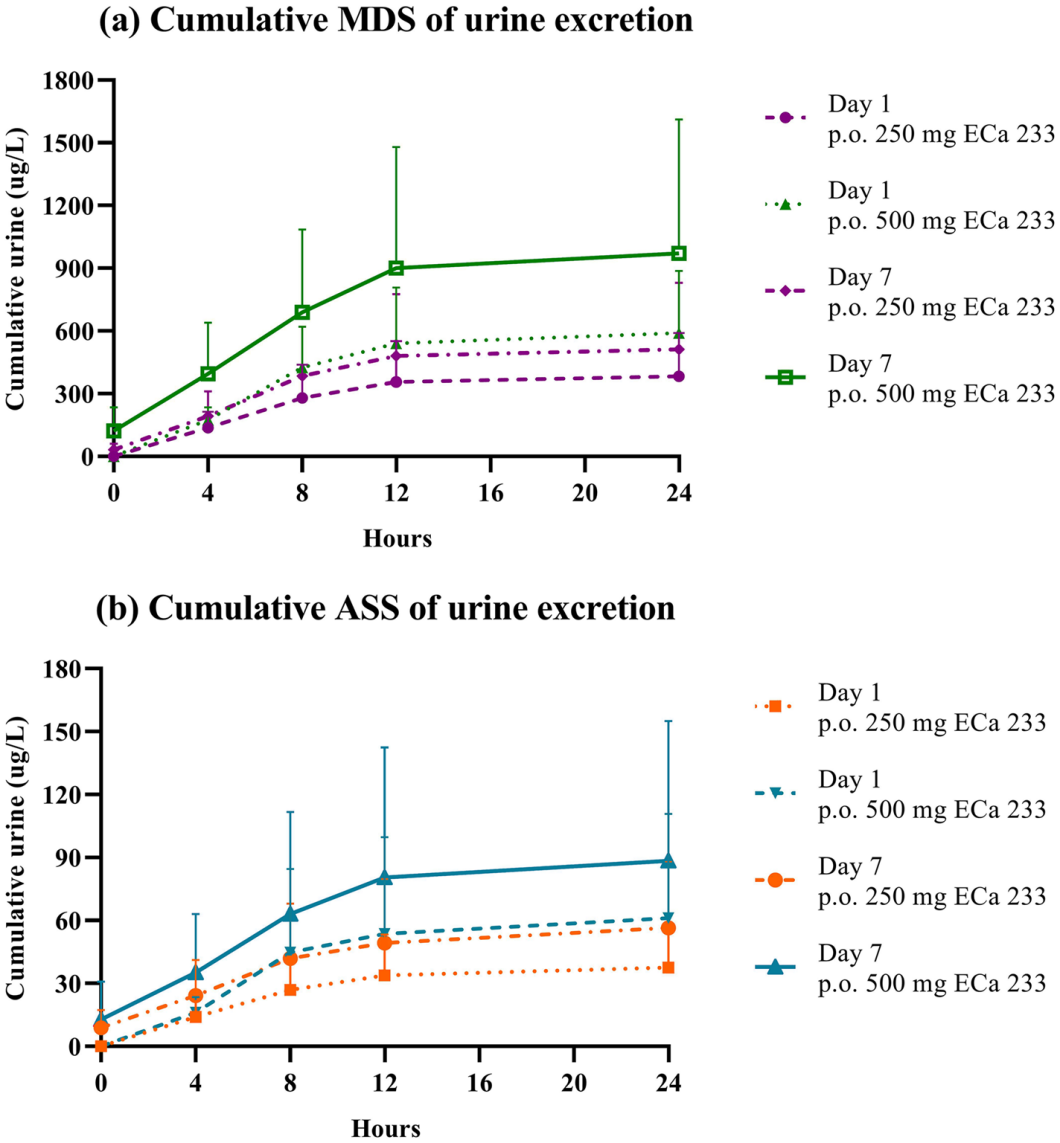
Cumulative urine excretion of (**a**) madecassoside and (**b**) asiaticoside after single or multiple oral administration of 250 or 500 mg doses in healthy Thai volunteers. Data are presented as means ± SD (n = 11–12).

The unchanged forms of MDS and ASS in plasma were primarily eliminated into the urine from the first 4 h until 24 h after dosing (Fig. 4). The highest cumulative contents of MDS and ASS were within 8 h, where only a small amount of the unchanged compounds was eliminated through the kidneys during 12–24 h. The active metabolites (MDA and ASA), however, did not follow the same pattern observed for their respective parent compounds, as evidenced by the absence of the metabolites in the urine. Fecal samples collected from one of the volunteers also corroborated this assumption, as MDA and ASA were both found in the feces.

### Metabolomics

#### Human metabolomic profiling

**Figure 5:**
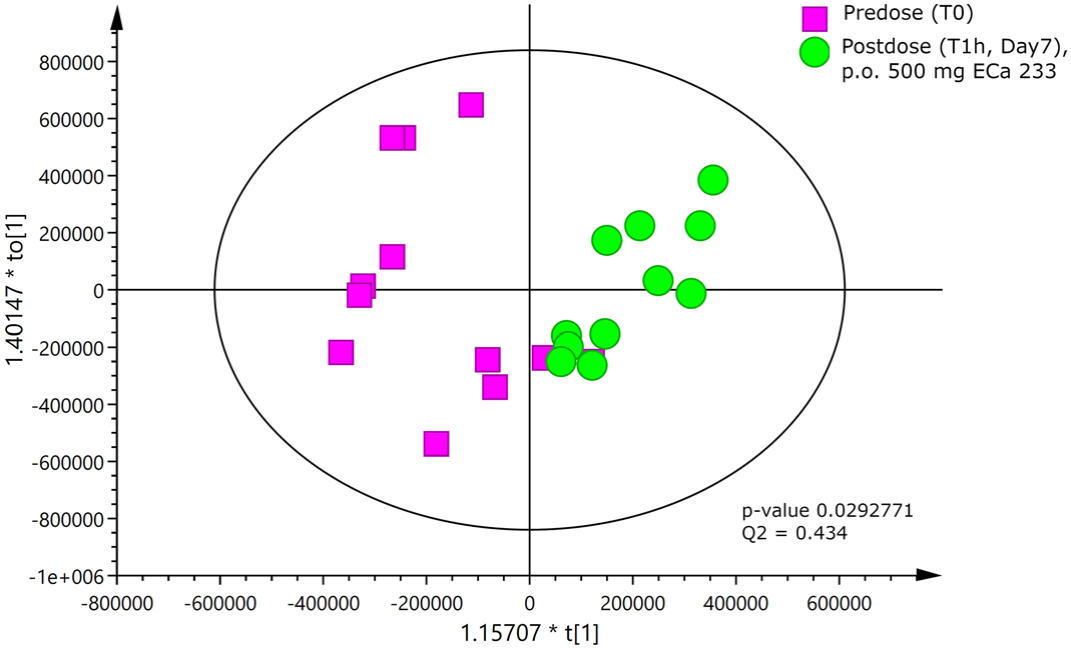
OPLS-DA score plot of human endogenous metabolites between the two clusters, pre- and post-dose, for repeated administration of 500 mg of ECa 233.

Following oral administration of a once-daily dose of 250 mg or 500 mg of modified-formula ECa 233 for 7 consecutive days, metabolomic profiling detected a total of 31 endogenous metabolites from 1D ^1^H-NMR spectra. Twenty-nine metabolites in plasma were identified according to the Chenomx NMR and human metabolome database (HMDB; Supplementary Fig. 4S). Clustering between pre- and post-dose receiving the repeated 500 mg of modified-formula was clearly separated based on the OPLS-DA score plot, indicating the discrimination of metabolites observed among those two clusters (Fig. 5).

**Figure 6:**
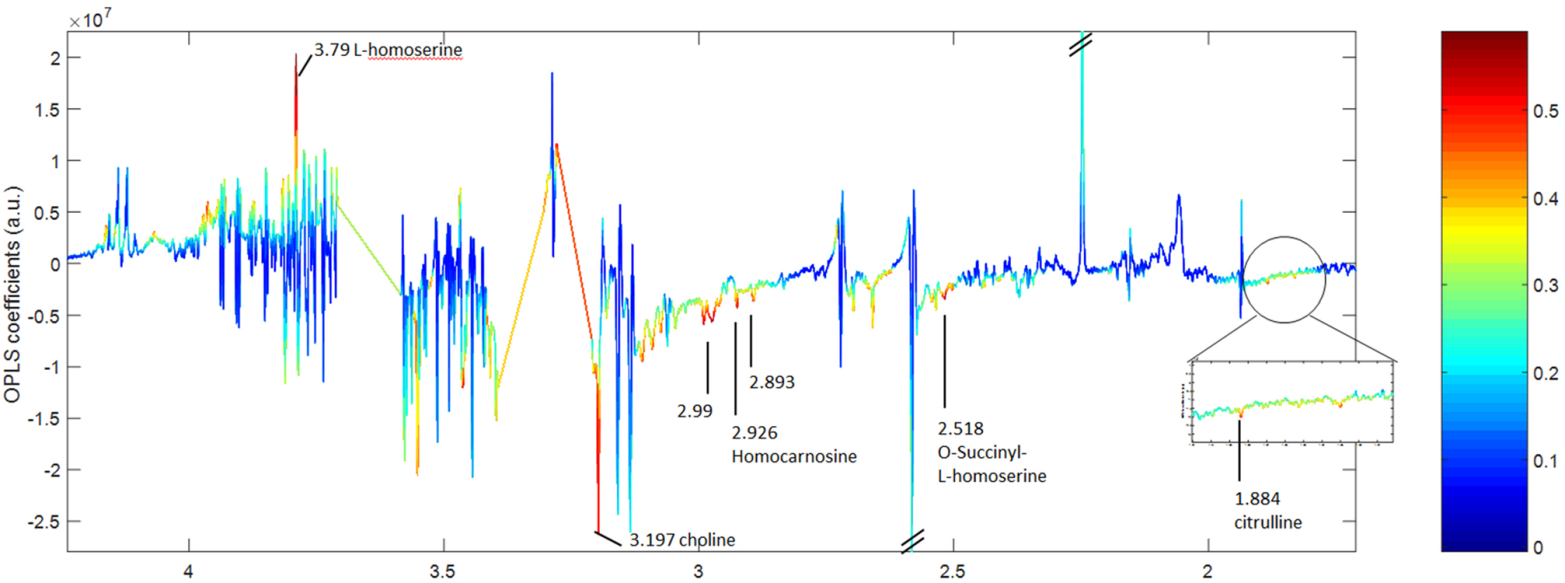
OPLS-DA coefficient loadings plot of different metabolites that were altered in the pairwise comparison (predose, T_0_ vs postdose, T_1h_). Resonances pointing upwards reflect metabolites that were greater in the pre-dose group relative to the post-dose group, whereas resonances pointing downwards indicate lower concentrations of metabolites in the pre-dose group relative to the post-dose group.

The results on OPLS-DA score plot suggested that a two-dose treatment (250 and 500 mg in multiple dose) had the same patterns of metabolome changes. The permutation p-value of the model validity was only significant for the group of 500 mg. Therefore, these findings may indicate a dose-dependent relationship in metabolomics. The OPLS-DA coefficient loadings plot was further used to investigate in the group of 500 mg repeated doses. The correlation of variables with classification of OPLS-DA models is shown in Fig. 6. Based on the results in the loadings plot, five potential metabolites (Table 3) were indicated as possible biomarkers for the metabolomics alterations related effects when taking repeated doses of 500 mg modified-formula ECa 233.

**Table 3:** Metabolic changes in human plasma and chemical shift of ^1^H NMR profiles (predose-T_0_ vs postdose-T_1h_ comparison). The validity of the models was evaluated using permutation p-values with R^2^X and Q^2^Y values.

Metabolite	Chemical shift	OPLS-DA models Predose (T_0_) vs MH (T_1h_)
		R^2^X = 93.63% Q^2^Y = 0.4861 Permutation *p-value* = 0.004
O-Succinyl-L-homoserine	1.85 (m); 2.06 (m); 2.47 (d); 2.52 (m); 3.65 (m); 4.24 (dd)	-0.7492
Homocarnosine	3.17 (dd); 3.21 (t); 4.48 (m); 7.01 (s); 7.90 (s)	-0.7315
Choline	3.21 (s); 3.52 (m); 4.07 (m)	-0.7114
L-homoserine	2.03 (m); 2.16 (m); 3.79 (m); 3.86 (dd)	+ 0.7645
Citrulline	1.58(m); 1.88(m); 3.15(t); 3.76(t)	-0.6722

( +) indicates metabolite that was higher in pre-dose group, whereas (−) indicates higher metabolites at post-dose (T_1h_) after multiple oral dosing of the modified ECa 233 at 500 mg. permutation p-value obtained from n = 1000 permutation tests.

*s* singlet, *d* doublet, *dd* double of doubles, *t* triplet, *m* multiplet, *m* multiplet.

The findings demonstrated the significant differences in levels of metabolites that varied in the pairwise comparison (predose T_0_ vs postdose T_1h_), (Fig. 6). In contrast, focusing on the metabolites at post-dose (T_1h_, day 7), these results showed that relative plasma concentrations of L-homoserine significantly decreased while levels of citrulline, O-succinyl-L-homoserine, homocarnosine, and choline were significantly increased after receiving modified formula ECa 233 in comparison to pre-dose levels at T_0_.

#### Metabolomics shifts

**Figure 7:**
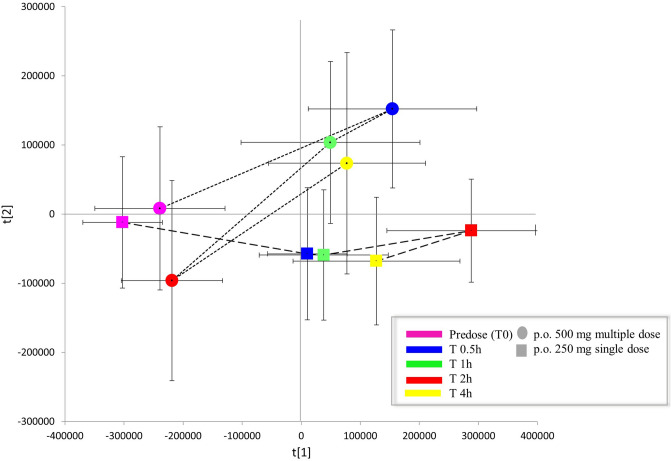
PCA trajectory scores plot of plasma data sets obtained from single low dose and multiple high dose; error bars are shown by standard deviation of the mean.

A shift in human endogenous metabolite profiles as a result of administered modified formula ECa 233 was observed between a single low dose (250 mg dose taken once) and multiple high dose (500 mg dose taken once daily for 7 days), (Fig. 7). The PCA trajectory scores plot between single low dose and multiple high dose at different time points (0, 0.5, 1, 2 and 4 h) revealed remarkable metabolic alterations. At pre-dose, the first time point demonstrates similar metabolic baselines of both groups suggesting that human endogenous metabolites at the beginning of the study are similar in both groups (Fig. 7). After receiving the modified formula ECa 233, distinct metabolic shifts were observed during 4 h at different magnitudes (Fig. 7).

### Safety and tolerability

The modified formula ECa 233 was well-tolerated throughout the study (Table 4). No serious adverse events were reported. The most commonly reported AEs were associated with the gastrointestinal system; mild abdominal discomfort was occasionally (n = 2/12) found during the 250-mg multiple oral dosing, and moderate flatulence (n = 1/11) with constipation (n = 1/11) were rarely reported in the group that received 500 mg doses. One participant reported serious drowsiness after receiving the multiple high doses of ECa 233; this AE was also included in the previous report after oral administration with high doses of *C. asiatica*^14^.

**Table 4:** Summary of safety and adverse events of the modified-formula ECa 233 after single and multiple oral administration in different dosage regimens.

Adverse events	250 mg (n = 12)		500 mg (n = 11)		Relation to medication
	Single n/12 (%)	Multiple n/12 (%)	Single n/11 (%)	Multiple n/11 (%)	
Drowsiness	3/12 (25%) Mild-moderate	3/12 (25%) Mild-severe	3/11 (27.27%), Mild-severe	1/11 (9.09%) Severe	Unlikely
**Gastrointestinal disorder**					
Nausea	0	1/12 (8.33%) Mild	0	0	Possible
Vomiting	0	0	0	0	–
Burning stomach	0	0	0	0	–
Abdominal discomfort	0	2/12 (16.67%) Mild	0	0	Probable
Flatulence	0	1/12 (8.33%) Mild	1/11 (9.09%) Moderate	1/11 (9.09%) Moderate	Probable
Increased urinary frequency	1/2 (8.33%) Mild	1/12 (8.33%) Moderate	0	0	1-possible 1-probable
**Others**					
Headache	1/12 (8.33%) Moderate	0	0	0	Unlikely
Constipation	0	0	1/11 (9.09%) Mild	1/11 (9.09%) Mild-moderate	Probable

## Discussion

This study found that daily doses of the modified formula ECa 233 was safe and appropriate for clinical uses. This newly developed ECa 233 capsule causes changes not only in disposition kinetics but also in endogenous human metabolomics profiling.

In comparison with the previous formulation^7^, AUC and C_max_ of MDS and ASS exhibited the improved absorption kinetics of this modified-formula, which indicates an increased solubility of the parent compounds. This can be explained by the formula’s enhanced dissolution from the addition of a small amount of solubilizer, an excipient that increases the bioavailability of pharmaceutical ingredients. The higher dissolution of this formula could lead to the increased MDS and ASS absorption and result in the improved ECa 233 oral bioavailability. Interestingly, a significantly greater systemic exposure was clearly seen in their respective metabolites (MDA and ASA). These results reflect a complete biotransformation of the parent compounds, the unabsorbed triterpenoid glycosides that can be completely hydrolyzed to become their respective metabolites (triterpenic acids). This process is carried out by an enzyme produced by intestinal bacteria that has the role of hydrolyzing glycosides^15^. In addition, the previous study reported the possible hydrolysis mechanism of ASS. This mechanism involves the hydrolysis of the sugar moiety of the glycoside (ASS) under acidic conditions, converting ASS into aglycone (ASA)^16^, which becomes the active component of *C. asiatica* extract that has pharmacological effects^17^. This modified formula ECa 233 had greater accumulation of active metabolites as seen in the increased AUC and C_max_ of ASA (*p* < 0.05), corresponding to the increased amount of ECa 233 in the administered dose. This accumulation supports the role of ASA in inhibition of P-glycoprotein (P-gp)^18^, an efflux membrane transporter that limits cellular uptake of xenobiotics. As a consequence of inhibiting P-gp expression, ASA (a substrate of P-gp) increases, then accumulates in the systemic circulation^19^. Other studies have also found an accumulation of ASA after oral administration of *C. asiatica* extract^20^.

In regard to the excretion behaviors, the study findings fill a gap in the knowledge around ECa 233. MDS and ASS are rarely absorbed but rather undergo extensive biotransformation with minimal renal excretion. Their active metabolites, MDA and ASA, which are highly lipophilic molecules, were likely eliminated through the hepatobiliary system and then mainly excreted by the fecal route. The results suggest that both major parent compounds were excreted mainly as metabolites via the feces. This is supported by the findings of other research in elimination pathways of *C. asiatica* extracts, which indicates that the excretion (0–72 h) of MDS was 0.25% in urine and 24.68% in feces^21^. Another study showed that the extract was excreted in feces over 24–76 h, while only a small amount of extract was eliminated via the kidneys^20^.

The accumulation behavior of the modified formula ECa 233 affected human metabolomic profiles, partly through an alteration of endogenous metabolites detected in plasma between pre- and post-dose. The changes in 5 relevant human metabolites were thoroughly considered to identify the candidate biomarkers; including L-homoserine, citrulline, O-succinyl L-homoserine, homocarnosine, and choline. In particular, increasing doses of ECa 233 to a 500-mg multiple oral dosing had a significant effect on increasing levels of choline, an endogenous metabolite reported to have benefits for learning and memory. It has been reported that higher concentrations of choline are believed to slow the progression of cognitive impairment^9^. The previous studies have suggested that a possible mechanism by which *C. asiatica* enhances cognitive function is through inhibition of acetylcholinesterase activity (AChEI). The studies also demonstrated this extract’s protective effect against ß-amyloid formation. The results indicated that ECa 233, an extract from of *C. asiatica*, might be considered as an AChEI alternative and responsible for increasing level of choline. Choline is a precursor of acetylcholine, a neurotransmitter that serves the neural activities in the learning and memory system. Furthermore, the reduction of AChE activity in cholinergic neurons is also involved with cognitive impairment as detected in Alzheimer’s disease^22,23^. This finding in healthy volunteers suggests that the recommended dose of ECa 233 should not be less than 500 mg/day in future studies investigating metabolite changes or exploring metabolic pathways related to cognitive deficits in patients.

Metabolite shifts were observed over 4 h after administration of multiple doses of ECa 233 (Fig. 7). The results indicated that changes of metabolomic profiling could be obviously detected over 1–2 h post-dose. This interval time is also considered as T_max_ in the pharmacokinetic results (T_max_ = 1–1.5 h, Table 2). These findings could imply that the relationship between drug disposition kinetics and the changes in human endogenous metabolites are affected by time after administration of high oral doses of ECa 233. Lastly, at 4 h later, the metabolic features had returned to nearly the starting point or closely to the initial metabolites again (Fig. 7), presumably because of the decreased concentration of the parent compounds and their active metabolites in plasma (Fig. 3). This pattern at end points conformed with the termination phase of ECa 233 as reflected in its excretion.

## Conclusion

This study presents the first reported evidence on the elimination kinetics of standardized extract of *C. asiatica* (ECa 233), indicating that the unchanged forms (MDS and ASS) are excreted partly through renal elimination. Fecal excretion is assumed to be the major pathway for their active metabolites (MDA and ASA). The enhanced-formula ECa 233 indicated higher absorption, which significantly increased its bioactive metabolites in healthy volunteers. This study reveals that administering a daily dose of at least 500 mg of ECa 233 can alter human metabolic profiling. Five human endogenous metabolites (L-homoserine, citrulline, O-succinyl-L-homoserine, homocarnosine, and choline) should be further investigated as candidate biomarkers in a phase II clinical trial.

## Supplementary Information

Supplementary Information.

## Abbreviations

AChE: Acetylcholinesterase
AChEI: Acetylcholinesterase inhibitor or cholinesterase inhibitor
AEs: Adverse events
ASA: Asiatic acid
ASS: Asiaticoside
AUC: Area under plasma concentration–time curve
BMI: Body mass index
Cl/F: Apparent total clearance of the test substance removed from plasma after oral administration
C_max_: Maximum plasma concentration
CPMG: Carr-Purcell-Meiboom-Gill pulse
ECa 233: Standardized extract of *Centella asiatica*
ESI: Electrospray ionization
F: Absolute oral bioavailability
HMDB: Human metabolome database
HPLC: High performance liquid chromatography
IQR: Interquartile range
IS: Internal standard
LC–MS/MS: Liquid chromatography tandem mass spectrometry
LLOQ: Lower limit of quantification
MDA: Madecassic acid
MDS: Madecassoside
MRM: Multiple reaction monitoring
NMR: Nuclear magnetic resonance spectroscopy
NOAEL: No observed adverse effect level
OPLS-DA: Orthogonal-signal correction-projection to latent structure-discriminant analysis
PCA: Principal component analysis
P-gp: P-glycoprotein
p.o. (Per os): Oral administration
SD: Standard deviation
STOCSY: Statistical total correction spectroscopy
T_max_: Time to reach maximum plasma concentration
TSP: Trimethylsilylpropanoic acid
t_1__/__2_: Elimination half-life
V_d_/F: Apparent volume of distribution after non-intravenous administration
