# Publisher Correction: Pharmacokinetics and metabolomics investigation of an orally modified formula of standardized *Centella asiatica* extract in healthy volunteers

**DOI:** 10.1038/s41598-021-92113-2

**Published:** 2021-06-09

**Authors:** Phanit Songvut, Pajaree Chariyavilaskul, Phisit Khemawoot, Rossarin Tansawat

**Affiliations:** 1 Department of Pharmacology and Physiology, Faculty of Pharmaceutical Sciences, Chulalongkorn University, Bangkok, Thailand; 2 Translational Research Unit, Chulabhorn Research Institute, Bangkok, Thailand; 3 Clinical Pharmacokinetics and Pharmacogenomics Research Unit, Department of Pharmacology, Faculty of Medicine, Chulalongkorn University, Bangkok, Thailand; 4 Chakri Naruebodindra Medical Institute, Faculty of Medicine Ramathibodhi Hospital, Mahidol University, Samut Prakarn, Thailand; 5 Preclinical Pharmacokinetics and Interspecies Scaling for Drug Development Research Unit, Chulalongkorn University, Bangkok, Thailand; 6 Department of Food and Pharmaceutical Chemistry, Faculty of Pharmaceutical Sciences, Chulalongkorn University, Bangkok, Thailand

Correction to: *Scientific Reports* 10.1038/s41598-021-86267-2, published online 25 March 2021

In the original version of this Article Phisit Khemawoot was incorrectly affiliated with ‘Department of Food and Pharmaceutical Chemistry, Faculty of Pharmaceutical Sciences, Chulalongkorn University, Bangkok, Thailand’. The correct affiliations are listed below.

Chakri Naruebodindra Medical Institute, Faculty of Medicine Ramathibodhi Hospital, Mahidol University, Samut Prakarn, Thailand

Preclinical Pharmacokinetics and Interspecies Scaling for Drug Development Research Unit, Chulalongkorn University, Bangkok, Thailand

Additionally, Rossarin Tansawat was incorrectly affiliated with ‘Chakri Naruebodindra Medical Institute, Faculty of Medicine Ramathibodhi Hospital, Mahidol University, Samut Prakarn, Thailand’. The correct affiliation is listed below.

Department of Food and Pharmaceutical Chemistry, Faculty of Pharmaceutical Sciences, Chulalongkorn University, Bangkok, Thailand

The original Article has been corrected.

