# Supplementary material for: Pharmacokinetics and metabolomics investigation of an orally modified formula of standardized Centella asiatica extract in healthy volunteers
Source: Sci Rep. 2021 Mar 25;11:6850. doi: 10.1038/s41598-021-86267-2 (PMC7994819; doi:10.1038/s41598-021-86267-2)
Supplement: Supplementary file 1 — Supplementary Information. [file 41598_2021_86267_MOESM1_ESM.pdf]

Pharmacokinetics and metabolomics investigation of an orally modified formula of standardized *Centella asiatica* extract in healthy volunteers

Phanit Songvut[1,2,#], Pajaree Chariyavilaskul[3,#], Phisit Khemawoot[4,5,*], Rossarin Tansawat[6,*]

Affiliation

[1]Department of Pharmacology and Physiology, Faculty of Pharmaceutical Sciences, Chulalongkorn University, Bangkok, Thailand

[2]Translational Research Unit, Chulabhorn Research Institute, Bangkok, Thailand

[3]Clinical Pharmacokinetics and Pharmacogenomics Research Unit, Department of Pharmacology, Faculty of Medicine, Chulalongkorn University, Bangkok, Thailand

[4]Chakri Naruebodindra Medical Institute, Faculty of Medicine Ramathibodhi Hospital, Mahidol University, Samut Prakarn, Thailand

[5]Preclinical Pharmacokinetics and Interspecies Scaling for Drug Development Research Unit, Chulalongkorn University, Bangkok, Thailand

[6]Department of Food and Pharmaceutical Chemistry, Faculty of Pharmaceutical Sciences, Chulalongkorn University, Bangkok, Thailand

[#]Equally contributing authors

***Corresponding Authors**

Rossarin Tansawat, Ph.D.

Department of Food and Pharmaceutical Chemistry, Faculty of Pharmaceutical Sciences, Chulalongkorn University, 254, Wang Mai, Pathumwan, Bangkok, Thailand

Tel.: +66 22188297

E-mail address: rossarin.t@pharm.chula.ac.th

Phisit Khemawoot, Ph.D.

Chakri Naruebodindra Medical Institute, Faculty of Medicine Ramathibodhi Hospital,

Mahidol University, Bang Phli, Samut Prakarn, 10540 Thailand

Tel.: +66 28395161

E-mail address: phisit.khe@mahidol.ac.th

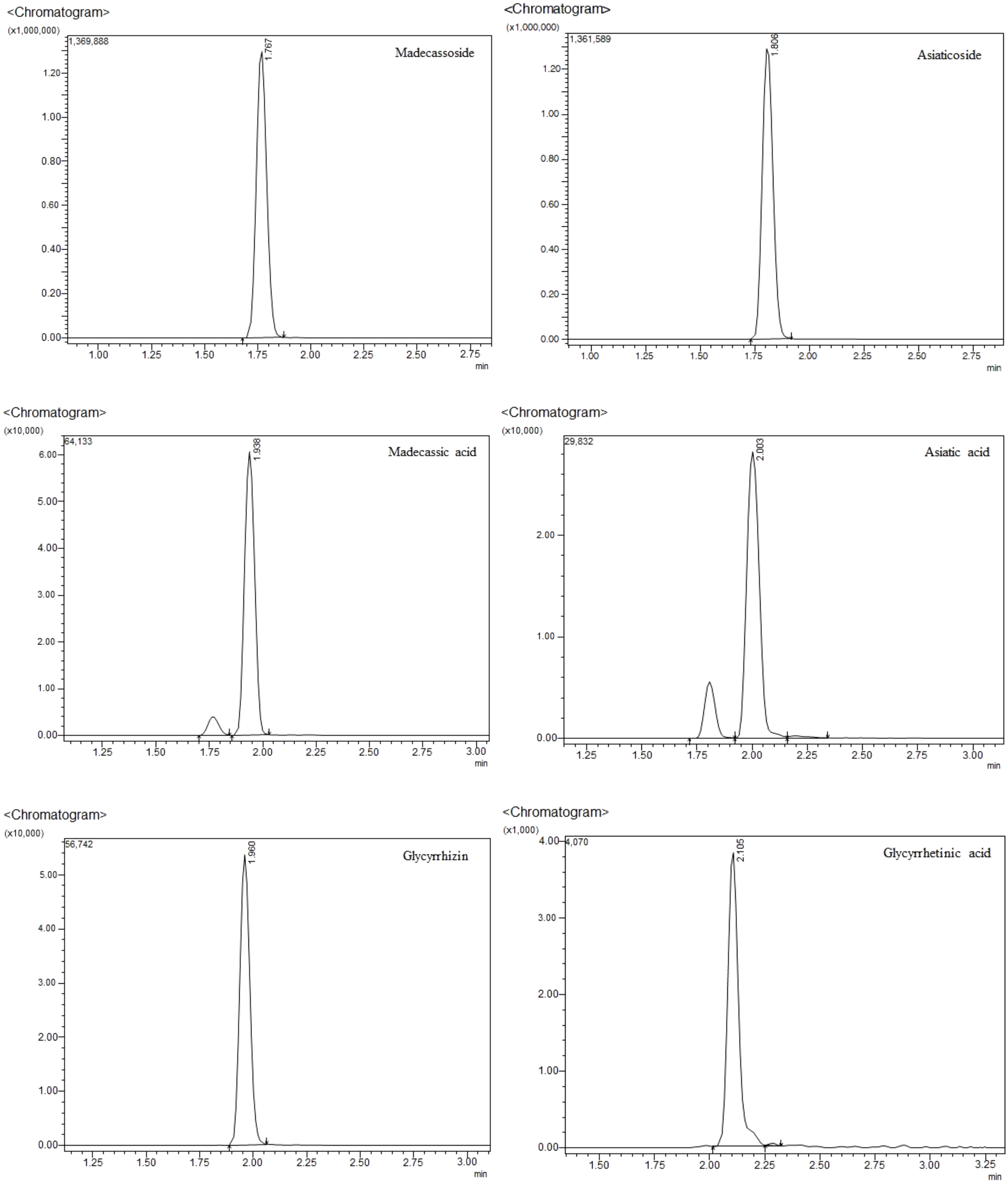

Figure 1S. Chromatogram and retention time of madecassoside, asiaticoside, madecassic acid, asiatic acid, glycyrrhizin, and glycyrrhetinic acid.

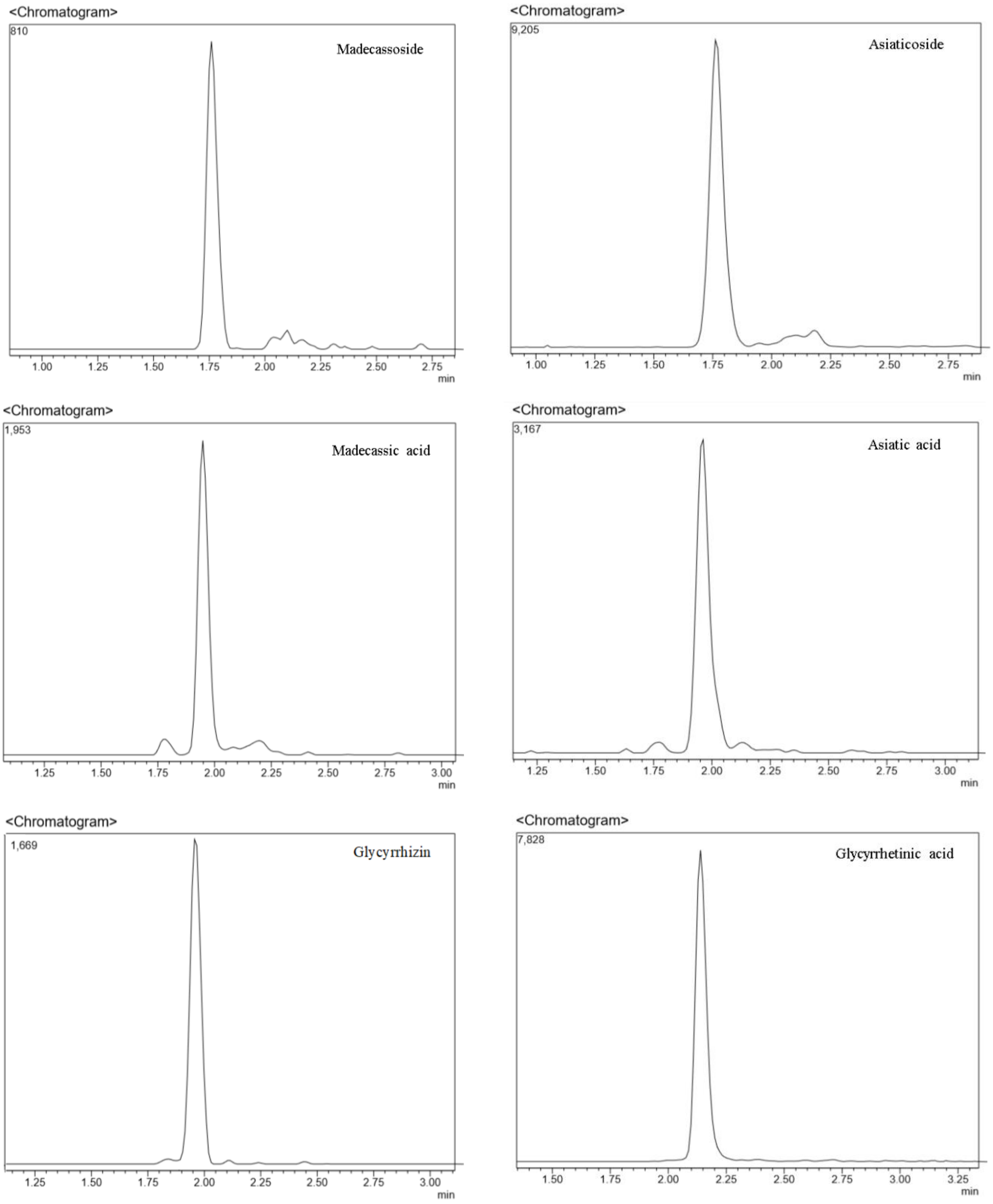

Figure 2S. Chromatogram and retention time in plasma.

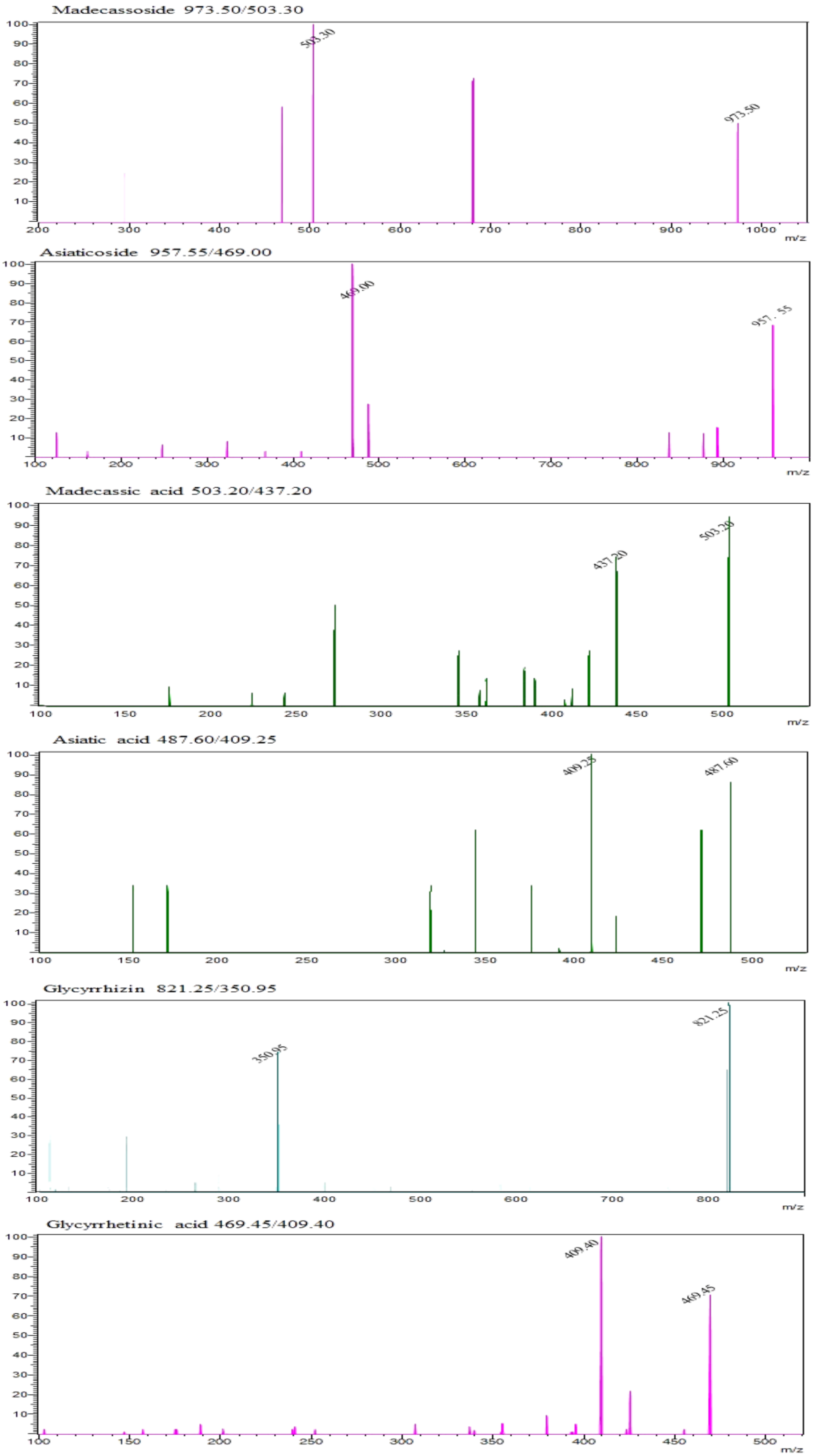

Figure 3S. Mass spectrums of madecassoside, asiaticoside, madecassic acid, asiatic acid, glycyrrhizin, and glycyrrhetinic acid

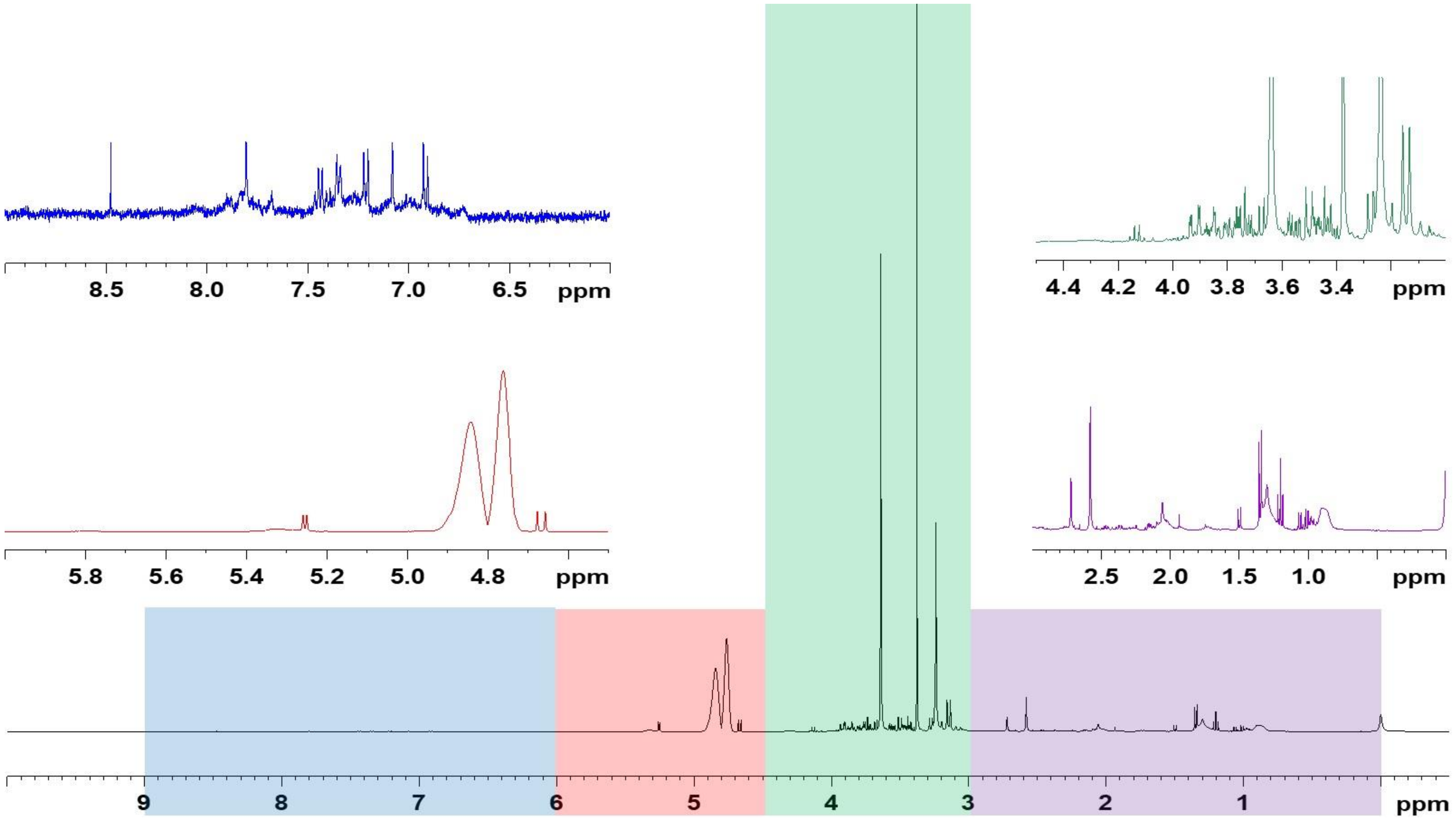

Figure 4S. NMR chromatogram
